# Risk prediction of clinical adverse outcomes with machine learning in a cohort of critically ill patients with atrial fibrillation

**DOI:** 10.1038/s41598-021-97218-2

**Published:** 2021-09-23

**Authors:** Lorenzo Falsetti, Matteo Rucco, Marco Proietti, Giovanna Viticchi, Vincenzo Zaccone, Mattia Scarponi, Laura Giovenali, Gianluca Moroncini, Cinzia Nitti, Aldo Salvi

**Affiliations:** 1 Internal and Sub-Intensive Medicine Department, A.O.U. “Ospedali Riuniti” di Ancona, Via Conca 10, 60126 Ancona, Italy; 2 Cyber-Physical Department, United Technology Research Center, Trento, Italy; 3 Department of Clinical Sciences and Community Health, University of Milan, Milan, Italy; 4 Geriatric Unit, IRCCS Istituti Clinici Scientifici Maugeri, Milan, Italy; 5 Liverpool Centre for Cardiovascular Science, University of Liverpool and Liverpool Heart and Chest Hospital, Liverpool, UK; 6 Neurological Clinic Department, A.O.U. “Ospedali Riuniti”, Ancona, Italy; 7 Emergency Medicine Residency Program, Marche Polytechnic University, Ancona, Italy; 8 Clinica Medica, Azienda Ospedaliero-Universitaria “Ospedali Riuniti”, Ancona, Italy

**Keywords:** Computational biology and bioinformatics, Cardiology, Medical research, Risk factors, Mathematics and computing

## Abstract

Critically ill patients affected by atrial fibrillation are at high risk of adverse events: however, the actual risk stratification models for haemorrhagic and thrombotic events are not validated in a critical care setting. With this paper we aimed to identify, adopting topological data analysis, the risk factors for therapeutic failure (in-hospital death or intensive care unit transfer), the in-hospital occurrence of stroke/TIA and major bleeding in a cohort of critically ill patients with pre-existing atrial fibrillation admitted to a stepdown unit; to engineer newer prediction models based on machine learning in the same cohort. We selected all medical patients admitted for critical illness and a history of pre-existing atrial fibrillation in the timeframe 01/01/2002–03/08/2007. All data regarding patients’ medical history, comorbidities, drugs adopted, vital parameters and outcomes (therapeutic failure, stroke/TIA and major bleeding) were acquired from electronic medical records. Risk factors for each outcome were analyzed adopting topological data analysis. Machine learning was used to generate three different predictive models. We were able to identify specific risk factors and to engineer dedicated clinical prediction models for therapeutic failure (AUC: 0.974, 95%CI: 0.934–0.975), stroke/TIA (AUC: 0.931, 95%CI: 0.896–0.940; Brier score: 0.13) and major bleeding (AUC: 0.930:0.911–0.939; Brier score: 0.09) in critically-ill patients, which were able to predict accurately their respective clinical outcomes. Topological data analysis and machine learning techniques represent a concrete viewpoint for the physician to predict the risk at the patients’ level, aiding the selection of the best therapeutic strategy in critically ill patients affected by pre-existing atrial fibrillation.

## Introduction

Atrial fibrillation (AF) is a common arrhythmia that can often concur to complicate the clinical course of patients admitted for a critical illness: in this specific population, AF can be observed in up to 33% of the admitted subjects^1^. As largely known, patients with AF have an increased risk of adverse outcomes, such as thromboembolic events, major bleeding (MB), cardiovascular and all-cause death^2^. Nowadays, the baseline assessment of thromboembolic and bleeding in the routine management of clinically stable AF patients represents a pivotal step for all the major international guidelines^3^. Notwithstanding, the management of AF in the critically ill patient is still object of debate^4^, being both thromboembolic and hemorrhagic risk difficult to be assessed for several confounding factors, as coagulation abnormalities, platelet number and function alterations, drug therapies, drug-drug and drug-pathology interactions which can occur in those patients.

While both CHA_2_DS_2_-VASc and HAS-BLED scores are almost universally recognized as the mainstay for the baseline evaluation of the “usual” AF patients, observational data suggest that the predictive ability of such scores is extremely limited in critically ill patients, due to the complex clinical status and to the overall clinical severity^5^. Despite this, and in absence of specifically-designed risk scores, stratification with CHA_2_DS_2_-VASc and HAS-BLED is still recommended by experts also in critical illness^6^. This is in line with other indications for the AF management in an emergency care setting observed in several guidelines^4^. However, since the in-hospital occurrence of thromboembolism or bleeding in a critically ill patient could radically modify the prognosis^7^, more accurate solutions are required to correctly stratify the risk of this specific category of subjects.

Acutely ill patients are often admitted from emergency departments (ED) to critical care facilities in presence of declining clinical conditions. Older subjects affected by several comorbidities and one or more acute organ compromise are often admitted to intermediate care or stepdown unit (SDU) beds. This specific population is burdened by older age, a relatively higher number of chronic organ insufficiencies and a high prevalence of pre-existing AF, which is often already treated according to current guidelines. In this setting, a good score should predict the requested outcome by considering not only the baseline risk, which can be evaluated with the already validated tools but also additive thromboembolic and hemorrhagic factors, as the acute pathologies leading to the hospital admission, blood count and coagulation abnormalities, procedures and therapies used to treat the critical illnesses. All these data can now be easily obtained with the increasing technological implementation of the emergency care system (defined as emergency department, subintensive care units and intensive care units), making this environment perfect for big-data collection. Medical information, such as demographic and clinical data, pharmacological therapy, physiological signs, laboratory analysis and radiologic results can be easily collected bedside and shape big and heterogeneous datasets^8^. Classical statistical methods combined with topological data analysis (TDA) can be used to explain relationships between variables in large and *complex* datasets, especially in critical biomedical and medical phenomena. In this context, *complex* means that the phenomena under analysis cannot be reduced to the identification of binary correlation among the clinical variables, but it shall account for n-ary correlation. TDA has been experimentally applied in medical studies regarding cancer^9^ and pulmonary embolism^10^.

### Aims

The main objective of this work is to train newer ML-based prediction models to predict the main AF-related outcomes in the critically ill patient: specifically, we present a data-driven experiment to predict therapeutic failure (defined as in-hospital death or ICU transfer), stroke/TIA and MB in critically ill patients affected by pre-existent AF. The decision to use TDA as a tool for data investigation and feature selection technique shall be rooted in the complexity of the cohort of critically ill patients reported in this paper. For the sake of completeness, the cohort under analysis contains patients with several degrees of criticality because of both their comorbidities and clinical history. In such complex cohorts, the events such as therapeutic failure (TF), stroke/TIA and MB cannot be modelled as functions of a given fixed set of variables. Thus, there is the need to identify for each cohort the right set of variables. In addition, the modelling phase shall consider that these patients might show conditions that are caused by the simultaneous occurrence of multiple other conditions. In this view, TDA is a suitable tool to identify higher dimensional correlations. This decision is supported by several papers that have been published in the last decade^11–17^.

## Patients and methods

The study was approved by the institutional review board named CERM (Comitato Etico Regione Marche), Prot. 168/2018, June 21st, 2018. The written informed consent to the use of personal data for research purposes was required for all the subjects admitted to the hospital. All patients were treated according to the clinical guidelines current at the moment of the hospital admission. AFICILL (Atrial Fibrillation In Critically ILL) is a retrospective cohort study enrolling medical critically ill patients affected by pre-existing AF admitted to the SDU of the internal and sub-intensive medicine department of the Azienda Ospedaliero-Universitaria “Ospedali Riuniti”, Ancona, Italy. Full details regarding the data collection procedure are reported in a previous paper^5^: we retrospectively considered a cohort of critically ill patients with pre-existing AF admitted to the internal medicine department of the Azienda Ospedaliero-Universitaria “Ospedali Riuniti”, Ancona, Italy. This department implemented, since January 01st 2002, an electronic medical record system (eMRS) for inpatients’ management. Discharge diagnoses are encoded according to ICD-9-CM. Thus, we selected all consecutive patients admitted with a concurrent AF diagnosis (ICD-9: 427.31) in the timeframe January 01st 2002–August 03rd 2007. This timeframe was chosen to optimize data collection and obtain a homogenous population in terms of clinical management and antithrombotic drugs use since all the patients were classified and treated according to one single guideline^18^. Moreover, the absence of direct oral anticoagulants allowed us to achieve a population with a similar stroke/TIA and MB risk. We then evaluated every single patient analyzing all the information in the discharge report. The anonymized dataset is publicly available^19^. The risk stratification was performed adopting the data available for each patient and collected on the day of admission in our department. Additionally, since this was a retrospective study and both haemorrhagic and ischemic risk could change rapidly during a critical illness, we have calculated the global patient’s risk by considering the AF-related therapies and procedures performed during the whole hospital admission. Further implementations of our system should be able to evaluate the modifications of haemorrhagic and thrombotic risk in real-time, allowing the clinician to reassess the patient when certain clinical conditions change during the critical illness. The main study outcome was the therapeutic failure (TF), defined as the composite of death that occurred during SDU admission or transfer to ICU due to the worsening of clinical conditions, requiring more intensive and invasive management according to the clinical evaluation of the attending physicians. Occurrence of concurrent clinical events during the SDU admission was also reported, with a specific interest in incident stroke or transient ischemic attack (stroke/TIA) and MB according to the ISTH definition^20^. To predict TF, stroke/TIA and MB with validated scores, we calculated, respectively, the APACHE-II score, the CHA_2_DS_2_-VASc score and the HAS-BLED score according to their original definitions^21–23^.

### Methodology for data analysis

We adopted a methodology accounting for several steps: (1) data pre-processing; (2) topological methods for dataset visualization and features selection; (3) training of interpretable machine learning (ML) classifiers. The dataset contained both multiple clinical and target variables^19^. The process focuses on one target variable per time by dropping out the remaining ones. The first step was to delete columns with missing data, then categorical variables were transformed into dummy variables to increase the dimensionality of the dataset.

### Topological data analysis

We performed TDA for the three study outcomes (TF, stroke/TIA and MB) adopting the *Kepler Mapper*^24^ algorithm. Mapper needs raw data (or samples), a clustering method (DBSCAN) that returns the number of clusters, and a filter function computed on the cluster’s members, named *lens*, and the percentage of overlaps among bins. For the sake of clarity, DBSCAN is an unsupervised based method for grouping (i.e., clustering) points relying on a metric space. In the beginning, DBSCAN selects a sample and puts it into the first cluster. In the subsequent iterations, the algorithm identifies the points that are closed (i.e., the distance is below a given threshold) to the first sample. Thus, the algorithm looks for their respective neighbours that will be added to the first cluster. If the algorithm does not find new neighbours it selects another point from the dataset and repeats the previous procedure to build the second cluster, and so on^25^. We recall that this paper aims to investigate the reliability of CHA_2_DS_2_-VASc score and HAS-BLED for the cohort under analysis and not to judge the quality of the doctor’s final diagnosis, i.e., bleeding or thrombosis. To this end, we have used them as *lenses* for the construction of the Mapper graphs. These *lenses* should be able to highlight if the dataset can be immediately partitioned into independent subgroups (disconnected subgraphs) that would confirm that there are clusters of patients showing clinical characteristics that would make easier their detection. To interpret *Mapper* graphs, we have cross-referenced the values of the lenses with the actual outcomes, which are binary evaluations (negative/positive). For both the scores we have found that patients who have received the lowest or highest score values agree with the actual outcomes, respectively negative and positive. However, patients with intermediate score’s values do not correspond always to a specific actual outcome and can be misclassified. It means that intermediate scores are “grey areas”, and further analysis is needed to overcome the uncertainty. We provide a more extensive description of this method in the S1File (Supplementary Methods). The new dataset was visualized using TDA and relevant topological structures were compared with statistical tests. The output of the statistical analysis was used for selecting relevant features.

### Interpretable machine learning

We last divided the dataset into a training and a test set, respectively with 70% and 30% of the samples. The training set was fed into a ML algorithm trained with automatic parameters tuning and k-fold cross-validation, i.e., k = 10, to achieve the highest accuracy. In this paper we have trained *XGBoost*, which is one of the most popular ML algorithms, regardless of the type of prediction task, either regression or classification, and is deemed to provide better solutions than other systems, becoming the "state-of-the-art” ML algorithm when dealing with structured data. *XGBoost* is a decision-tree-based ensemble ML algorithm that uses a gradient-boosting framework^26^. Since its introduction, *XGBoost* has not only been credited with winning numerous competitions but also for being the driving force under the hood for several cutting-edge applications. For the love of completeness, in the past we have challenged other ML techniques in the same dataset^27^. However, generally, those techniques are not suited for interpretability, and they are considered “black boxes”. In this paper, we have enhanced the request to have interpretable methods toward personalized diagnosis and patients’ management as we did in other studies^9^. To achieve the highest accuracy, we have combined the *Scikit Learn Pipeline* and *GridSearch* methods to select the best hyper-parameters for each *XGBoost* instance^28,29^. With best hyper-parameters, we meant the assignment of the parameters such that they maximize the classifier’s accuracy as reported in the confusion matrix. In this paper, we have grid-searched both the number of estimators [5, 10, 50, 100, 200, 300, 500] and the max depth: [5, 10, 15, 20, 25]. In addition, to reduce the risk of overfitting, we have implemented the early-stopping strategy with a grid-search approach over hyper-parameters^30,31^. The performances of the trained algorithm were evaluated on the test set and reported by confusion matrix and by Area Under Curve (AUC) Receiver Operating Characteristic Curve (ROC). The McNeil method^32^ was used to test the statistical significance of the difference between the AUCs. Tools for ML models’ interpretation developed by information theory can detect the presence of any biases in the trained model. The same tools are used to pinpoint the relevance of each input feature referring to the trained model. To complete the interpretation of ML outputs, we have adopted interpretability methods. In the context of ML, interpretability means the ability to explain and validate the decisions of a predictive model to enable fairness, accountability, and transparency in algorithmic decision-making. In addition, the interpretation shall be provided in a human-readable format. Ideally, interpretation should be able to support the users, i.e., doctor and patient, to understand the “what, why, and how” of the ML behavior^33^.

## Results

We defined and performed the analysis on a final dataset of 1326 patients regarding the three study outcomes (TF, stroke/TIA and MB), originally described by 46 clinical variables. The full dataset is available in Mendeley Data repository^19^. A synthesis of the database structure is reported in Table S2. Originally, 1705 consecutive patients with pre-existing AF were evaluated^5^. After excluding those admitted for an elective cardioversion procedure and patients complicated by trauma (excluded for an increased, non-AF-related bleeding risk), we obtained a cohort of 1326 patients. We observed a total of 188 (14.1%) TF, with 152 deaths and 36 ICU transfers. After the SDU admission, 199 (15.0%) patients developed stroke/TIA while 140 (10.6%) complicated their clinical course with MB. In the selected cohort of patients, the median of APACHE-II score was 16 [4], the median of CHA2DS2-VASc was 4 [2] and the median of HAS-BLED was 2 [1].

### Therapeutic failure

**Figure 1:**
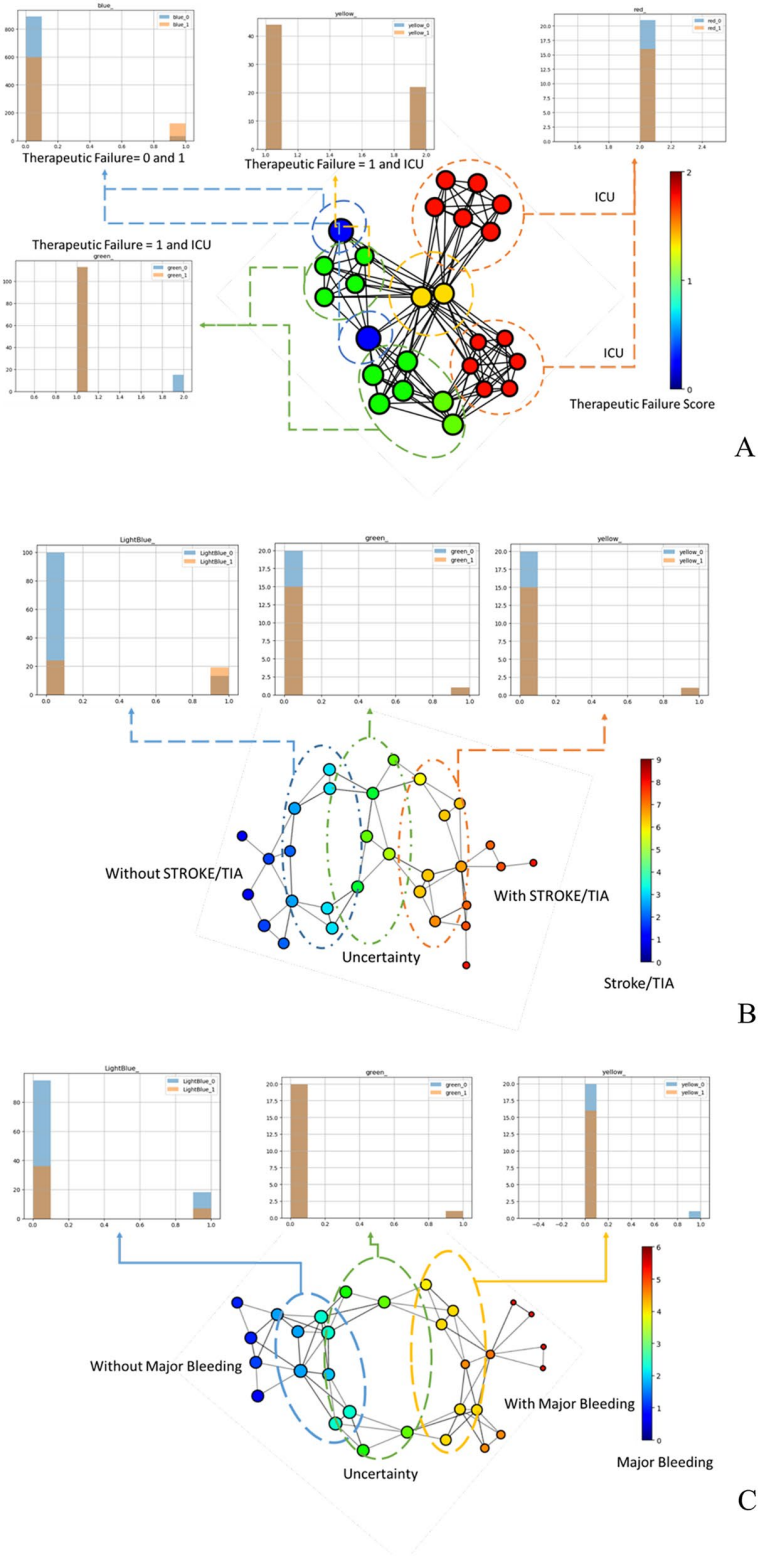
Topological data analysis results for (**A**) “therapeutic failure”; (**B**) “stroke/TIA” and (**C**) “major bleeding”. This output figure has been generated with Kepler Mapper 2.0.1^24^. Legend: ICU = intensive care unit.

The analysis of the topological graph for the TF outcome (Fig. 1, Panel A) highlights that the patients labelled as “ICU transfer” cluster into two specific subgroups (red nodes). However, some of the patients labelled as “ICU transfer” have also some similarities with patients labelled as “in-hospital death” (yellow nodes). The patients included in the “yellow” groups are also connected to those included in the “blue nodes” and “green nodes” groups, which have a less easily characterizable risk profile. “Blue” and “green” patients are less easily detachable but, while they share several features, they do have a differential risk profile. The “green” group is characterized by patients with average age 83 ± 7 years, mean (± SD) systolic blood pressure (SBP) 98.09 ± 31.05 mmHg and diastolic blood pressure (DBP) 59.37 ± 16.81 mmHg and contains only patients not treated with angiotensin-converting enzyme inhibitors/angiotensin receptor blockers (ACEi/ARBs). All the patients of the “green” group have reported intravenous amine use and concomitant systemic infections. The average age for the “blue” group is 79 ± 9 years, with a mean (± SD) SBP 128.09 ± 25.43 mmHg and DBP 76.68 ± 14.23 mmHg. This group contains equally distributed patients with and without ACEi/ARBs. All the patients in the “blue” group were not treated with intravenous amine use and did not report concomitant systemic infections. Blue and green groups represent patients with clinical similarities but with different clinical outcomes.

### Stroke/TIA

The analysis of the topological graph related to the “stroke/TIA” event (Fig. 1, Panel B) reveals that there are only few patients who would have almost certainly experienced the event (*with stroke/TIA*, “dark-red” nodes) and who almost would not have certainly experienced the event (*without stroke/TIA*, “dark-blue” nodes), that do not share any similarities. Most of the patients shape the circular motifs in the plot, with variable ranging risk. Specifically, we highlight three main subgroups: the “light-blue” group contains patients with lower CHA_2_DS_2_-VASc scores (2–3). The nodes with a score equal to 3 are connected to the “green” nodes characterized with a medium–low score (4). The green nodes are connected to the nodes with medium–high scores (5–7).

### Major bleeding

The analysis of the topological graph related to the MB event (Fig. 1, Panel C) reveals that there are only a few patients who would almost certainly have experienced the event (*with MB*, “dark-red” nodes) and who would not have almost certainly experienced the event (*without MB*, “dark-blue” nodes) that do not share any similarities. Most of the patients are arranged in circular motifs, with variable ranging risk. Specifically, we highlight three main subgroups: the “light-blue” group contains patients with low HAS-BLED scores (1–2). The nodes with a score equal to 2 are connected to the “green” nodes characterized with a medium score (3). The “green” nodes are connected to the nodes with higher HAS-BLED scores (4–5).

### Topology-driven feature selection

To train a ML classifier to predict if a new unseen patient has the probability to experience a certain clinical event, it would be better if the classes (for example healthy and ill) used for the training are strongly separated, with no overlaps among samples. TDA underlined that the cohort is not naturally separated into independent subgroups. Thus, there is the need to detect the features that can improve the separation, dropping those variables which don’t allow to discriminate the risk. To this extent, we combined the TDA output with standard statistic tests. Specifically, we evaluated the dependency among the clinical variables and the target variable under modelling by performing the χ^2^ test with the Yates correction for continuity to evaluate the dependency among categorical features and the target variable. The F-value was used to study the dependencies of the discrete variables on the target variable. This procedure can be represented as follows: the algorithm takes as input all the clinical features and one clinical outcome at a time (TF, stroke/TIA and MB), then (1) Kepler Mapper is used to build a topological graph representing the dataset, (2) DBSCAN is used as clustering method, (3) The percentage of overlaps among bins were selected after different manual tests, (4) TF, CHA2DS2-VASc and HAS-BLED are used as lenses accordingly to the outcome under analysis, (5) relevant topological structures belonging to both positive and negative clusters are compared with statistical tests, (6) the output of the statistical analysis is used for selecting relevant features. The results of this analysis are in S3 Table, where we report only the features obtaining a *p* value < 0.05. This step is crucial to reduce the number of the original clinical variables by removing the ones that are not related to the target variables. The initial set contained 46 variables, the reduced adopted for the analysis accounted of: 19 clinical variables used by the ML model of the *Therapeutic Failure* target variable, 19 clinical variables needed by the ML model of the *Stroke/TIA* target variable and 15 clinical variables required by the ML model for the prediction of the *Major Bleeding* target variable*.*

### Machine learning classifiers

The features identified with the statistical tests were used as input for ML. Modelling of the *XGBoost* algorithm was executed by evaluating different combinations of the main parameters. To tame the unbalancing among classes in the dataset when splitting the dataset into a train (70%) and a test (30%) set, we have imposed an equal distribution of positive samples in both subsets. Moreover, we adopted a tenfold cross-validation to increase the reliability of the algorithm. Models’ performances were evaluated using the classification error, that is the percent of incorrect classifications, with a minimum possible score equal to 0 (S2 Fig). The performances of the selected pipelines are reported in terms of average AUC-ROC on the testing set and corresponding 95%CI*.*

**Figure 2:**
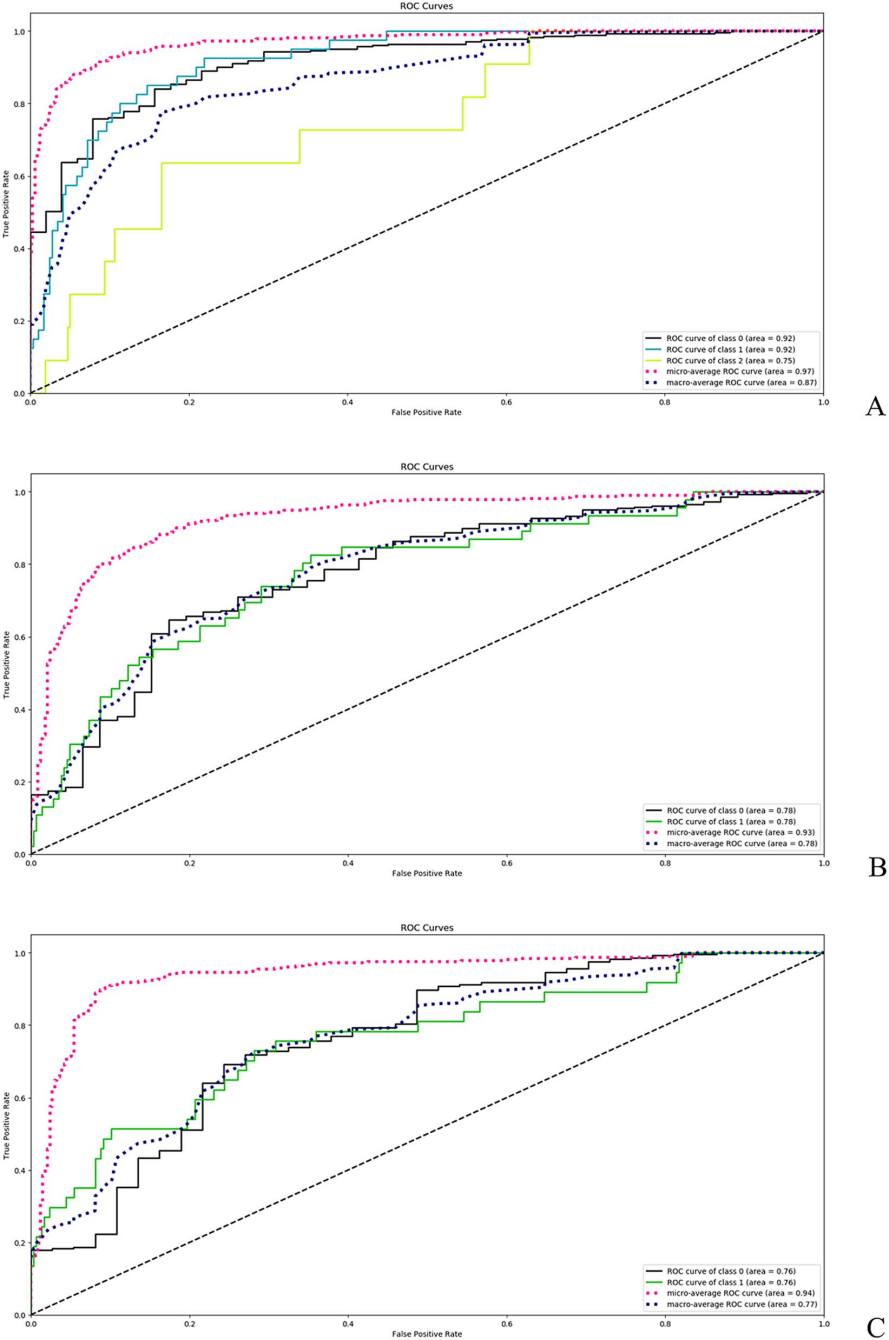
Predictive ability of machine-learning derived models for (**A**) “therapeutic failure”; (**B**) “stroke/TIA”; (**C**) “major bleeding”.

For TF, we have compared the ML-based score with the APACHE-II score. The APACHE-II was able to predict significantly the therapeutic failure or the transfer to ICU with an AUC of 0.953 (95%CI: 0.931–0.976). The ML-based solution for TF (best configuration: max-depth = 5 and number-of-estimators = 100) reached a slightly greater accuracy with an AUC of 0.974 (95%CI: 0.934–0.975, Fig. 2, Panel A; *p* < 0.0001 when comparing the two ROC curves). As previously reported for the same cohort under analysis^5^, the CHA_2_DS_2_-VASc score was not able to predict significantly the in-hospital occurrence of stroke/TIA (AUC:0.545;95%CI:0.489–0.601)^5^. The newly developed ML-based solution for stroke/TIA (best configuration: max-depth = 5 and number-of-estimators = 50) got an AUC of 0.931 (95%CI: 0.896–0.940; Fig. 2, Panel B; *p* < 0.0001 when comparing the two ROC curves, Brier score 0.13). Similarly, the HAS-BLED score was not able to predict significantly the in-hospital occurrence of MB (AUC: 0.503; 95%CI: 0.453–0.554)^5^. The newly developed ML scoring system for major bleeding (best configuration: max-depth = 5 and number-of-estimators = 50) outperformed the clinical score with an AUC of 0.930 (95%CI: 0.911–0.939, Fig. 2, Panel C; *p* < 0.0001 when comparing the two ROC curves, Brier score 0.09). Brier score for TF was not computed since this score was designed for dealing with only binary classifiers.

### Global machine learning interpretation

**Figure 3:**
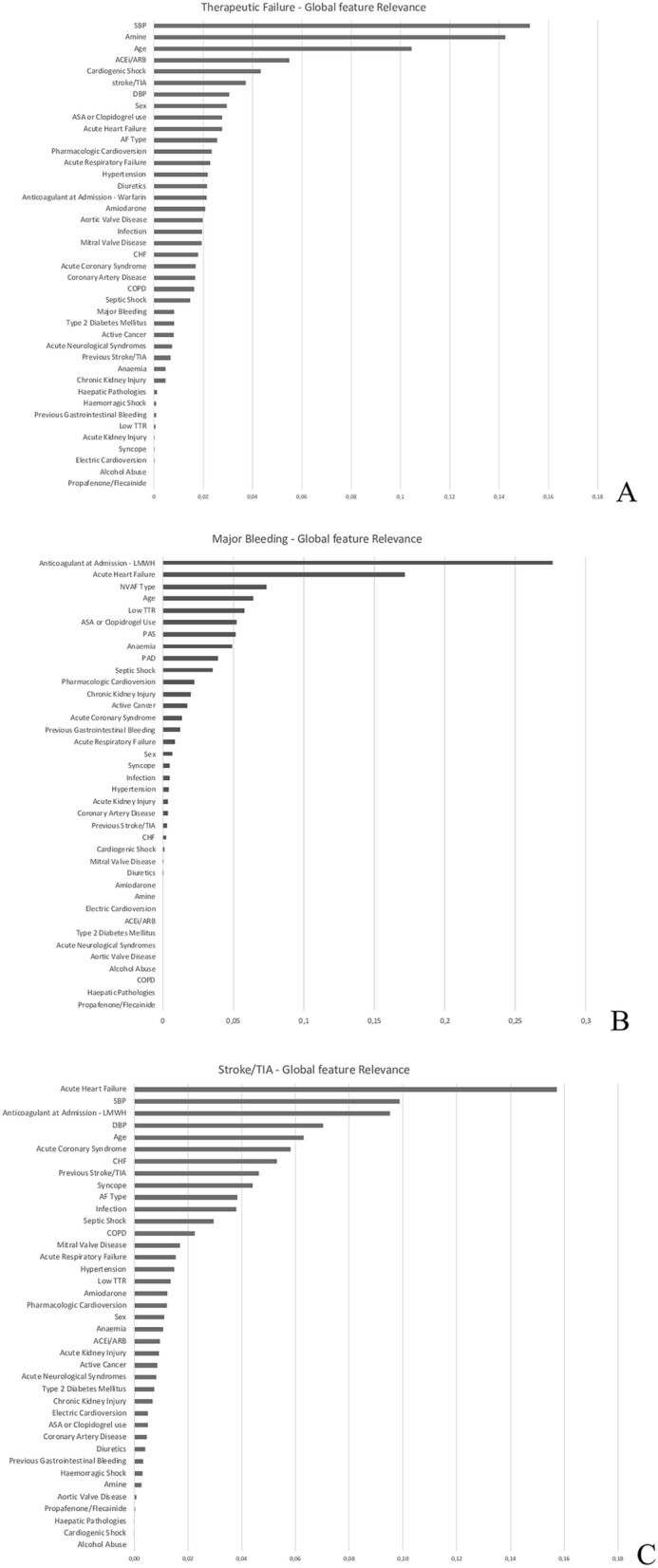
Skater results for (**A**) “therapeutic failure” (**B**) “major bleeding” (**C**) “stroke/TIA”.

The relevance of every single input variable for the trained ML model was computed by Skater. For the TF prediction model, the most important features were SBP, intravenous amine use and age. These variables reached an importance score between 0.15 and 0.10, followed by ACEi/ARB (0.05), cardiogenic shock (0.04) and stroke/TIA (0.04). In general, the features with less global impact were Propafenone/Flecainide use, alcohol abuse, electric cardioversion and gender, as shown in Fig. 3, Panel A. For the *stroke/TIA* prediction model, the most important feature was acute heart failure (AHF) with a score of 0.16, followed by SBP (0.10) and by the use of LMWH at admission (0.10). The variable and their relevance for the ML model trained to predict stroke/TIA are shown in Fig. 3, Panel B. For the *MB* prediction model, the most important features were the type of anticoagulant (LMWH) at the admission (0.21) followed by AHF (0.12). The features and their relevance for this machine model are shown in Fig. 3, Panel C. Less relevant variables cannot be discarded since they are relevant for the classification of single patients as underlined by Local Machine Learning Interpretation (LIME). In other words, even characteristics that contribute marginally to the global prediction can be discriminant for the single patient.

### Local machine learning interpretation

LIME allowed us to characterize every single patient’s risk starting from the features selected by the ML algorithm: by considering the same Skater variables, LIME assigns a specific, patient-dependent weight to each feature, which can be less relevant for the global model but discriminant for the single subject^9^. LIME’s plot interpretation is straightforward: given the probability P(C_i_) of a patient to be classified in one of the *i*th classes (e.g., P(C_1_) = 0.90, P(C_0_) = 0.10), by subtracting from P(C_i_) the weights of the variables characterizing the *i*-th class it is possible to compute what would be the new probability to belong to the current class or the others. Thus, LIME can give specific information regarding the individual risk of each analyzed patient calculated based on the global features. An example of LIME capabilities is described in the S1 File (Supplementary Methods). Experienced readers might doubt the choice of LIME instead of SHAP (SHapley Additive exPlanation). It is known that the former might be less accurate, but LIME is faster than SHAP^33^ The LIME histogram can be found in the supplementary material and they are depicted in the S3–S5 Figs. In addition, we remark that the accuracy of global machine learning interpretation is more important for the scope of this paper, which is the definition of new smart scoring systems for the diagnosis of the three target variables.

## Discussion

The critically-ill patient is considered at high risk of both haemorrhagic and thromboembolic complications for several, different mechanisms^34^: despite their increasing complexity, there are no specific indications on the management of these subjects, especially regarding anticoagulation, in the setting of critical care, mainly due to a lack of reliable clinical predictors of stroke/TIA and MB. Patients with pre-existing AF admitted for critical illness have a baseline thromboembolic risk which could be further raised by other factors related to the coexistent acute pathology, its treatment, organ dysfunctions and systemic inflammation^35^. CHA_2_DS_2_-VASc is a generic marker of risk, proven to be useful in several settings different from its original scope^36^. However, it is not validated for the critical illness, where it could be representative of the subject’s baseline risk, not considering the factors associated with the critical care environment. According to our results, CHA_2_DS_2_-VASc was not able to predict the individual risk to develop a stroke/TIA during the hospitalization and, consequently, to guide safely the patient’s management^5^. Our approach reached a good accuracy in predicting stroke/TIA during the hospitalization, sharing some items with CHA_2_DS_2_-VASc score, such as CHF, age, previous stroke/TIA and vascular disease, confirming the robustness of these features even in this setting. We also underlined the importance of some critical-care specific items, which could carry a major weight in the prediction of this outcome, such as admission diagnosis (i.e. AHF, ACS), physiologic parameters (SBP, DBP), and therapeutic management. We were also able to emphasize the role of some comorbidities, such as chronic lung and kidney disease, and the importance of the anticoagulant approach preceding the admission. Of note, both COPD^37^ and CKD^38^ have already been identified as adjunctive risk factors for stroke/TIA during AF but, despite their high prevalence, they are not included in commonly used risk scores.

Similarly, the HAS-BLED score did not show any accuracy in discriminating patients undergoing MB during the hospitalization in this cohort^5^. Again, the individual bleeding risk could be raised due to several factors which are commonly observed in the acute phase of a disease. Stress ulcers^7^, consumption of coagulation factors and reduction of platelet count are commonly observed in critical care. Several drugs, such as antiplatelets, are often needed in the acute phase of certain diseases, such as ACS, but can exponentially increase the bleeding risk, especially in presence of organ dysfunctions^39^.

Our method accurately predicted MB following hospitalization. Some HAS-BLED features such as age, anaemia, previous gastrointestinal bleeding, low TTR and antiplatelet drugs use were associated with MB, thus confirming the validity of these items even in the acutely ill patient. Comorbidities and specific factors for critical illness, such as the acute diseases leading to hospitalization, physiological parameters at the admission and the anticoagulant therapy were also associated with MB. We also engineered an accurate prediction model for TF, accounting for both general and disease-specific factors. Interestingly, both stroke/TIA and MB carried a major weight in the determination of this outcome, underlining the urgent need for specific models able to accurately predict thromboembolic and bleeding events in this setting to improve medical management and reduce in-hospital mortality.

Our paper also underlines a significant aspect related to the use of clinical scores in the management of AF patients: in the last years, several scores have been proposed to replace both CHA_2_DS_2_-VASc and HAS-BLED, with limited results in obtaining a significant improvement in prediction ability when tested outside the original validation cohorts^40^. A systematic review highlighted that most of the scores reported a similar predictive ability irrespectively of a larger number of items considered and differential use of weighting, with both CHA_2_DS_2_-VASc and HAS-BLED resulting among the most effective in determining the future outcomes risk^40^. A good clinical score is represented by the balance between evidence, practicality and robustness^41^: the results presented in this paper can illustrate that a more advanced analytical strategy can be useful to obtain a more accurate model, both considering a set of usual strong risk factors and more specific clinical characteristics. The emergency-care environment is becoming the ideal place to apply ML techniques in clinical practice, mainly due to its technological implementation: the wide use of electronic medical records, daily updated with drug therapy modifications, laboratory analysis data and physiological parameters allows the generation of large, dynamic datasets. The software integration of this data flow with ML algorithms will allow the clinician to easily obtain a real-time estimate of both thrombotic and bleeding risk. Moreover, the spreading use of mobile apps among physicians would allow a larger use and application of these methods. Notwithstanding, it’s important to underline how the clinical use of prediction models should assist and inform the clinical decision, rather than replace the clinical assessment and evaluation^41^.

## Study limitations

The main limitation is related to the study design, being a retrospective observational analysis of a cohort not primarily identified for research purposes. Some features, such as the time since the AF diagnosis, were not available and should be considered in further implementations of the model. Larger and external, multi-centre, prospective validations of these models will be required to confirm our results and to substantiate our methods. Moreover, since the thrombotic and the haemorrhagic risk of the critically ill patient changes dynamically as his pathology evolves, validation should be performed with a dynamically updated dataset, whose results would update daily the physician on the risks according to physiological parameters, laboratory analysis results, therapies and procedures performed. Moreover, it is necessary to underline that the deployment of such a solution in a real-life clinic might be unfeasible or at least feasible only in strongly-digitized countries: the implementation of such an algorithm in most hospitals, today, can be limited by technological, ethical and legislative barriers that could strongly limit the implementation of our approach in the clinical practice. Despite these limitations, currently several ongoing efforts are trying to solve these issues: for example, the European Commission has released a list of 7 requirements, ranging from ethical to technical indications, which could help to translate artificial intelligence projects into real-life applications^42^. Several authors, however, are investigating to solve legal, ethical and technical issues that might prevent the adoption of ML-based solutions in real-life situations^43^.

## Conclusions

In critically ill patients with pre-existing AF, the classical risk scores adopted to predict stroke/TIA and MB are not effective and should not be used to guide the therapeutic approach during a hospitalization into such a high level of clinical complexity. Big data analysis with TDA allowed us to identify specific risk factors associated with stroke/TIA and MB in this clinical setting. ML techniques were able to outperform classical risk scores. Moreover, in this paper we have also challenged tools to debug the ML models and understand the classification outputs. We believe this is a seminal step toward the instrumentation of a ML framework compliant with the GDPR-22nd article “right to be informed”.

## Supplementary Information

Supplementary Information.

## Data Availability

AFICILL Database is publicly available at: 10.17632/c87p293wpb.4 (DOI) or https://data.mendeley.com/datasets/c87p293wpb/4 (Mendeley Data).
