# Supplementary material for: Risk prediction of clinical adverse outcomes with machine learning in a cohort of critically ill patients with atrial fibrillation
Source: Sci Rep. 2021 Sep 23;11:18925. doi: 10.1038/s41598-021-97218-2 (PMC8460701; doi:10.1038/s41598-021-97218-2)
Supplement: Supplementary file 1 — Supplementary Information. [file 41598_2021_97218_MOESM1_ESM.docx]

**S1. SUPPLEMENTARY METHODS**

We adopted a methodology accounting of four steps: data pre-processing, topological dataset visualization, topology-driven feature selection and generation of interpretable machine learning (ML) classifiers. Topological data analysis (TDA) and feature selection performed with Mapper is synthesized in S1 Fig. We used a dataset containing both multiple clinical and target variables. The process focuses on one target variable per time by dropping out the remaining ones. The first step was to delete columns and rows with missing data, then categorical variables were transformed into dummy variables in order to increase the dimensionality of the dataset. The new dataset was visualized by means of TDA and relevant topological structures have been compared with statistical tests. The output of statistical analysis is used for selecting relevant features. Then, we divided the dataset into training and test set. Training set was fed into a ML algorithm trained with automatic parameters tuning and k-fold cross-validation in order to achieve the highest accuracy. The performances of the trained algorithm have been evaluated on the test set and reported by confusion matrix and other accuracy measurements in order to improve the interpretation of machine learning model, to reveal buggy training and test data and to express algorithm’s performance qualitatively and quantitatively. Training data can contain biases picked up by the model, reducing its performances in the test set. The presence of biased data could bias model evaluation: model interpretation can be used to debug disappointing performances in its engineering.

*Topological Data Analysis:* Topology is the branch of mathematics that aims to study shapes and maps: a topological space is an abstract space equipped with notions of similarities. A new set of algorithms for the construction of topological spaces and their analysis has been derived from algebraic topology, known as TDA, which is nowadays largely used for exploratory data mining. TDA can be derived in three main classes of algorithms: Persistent Homology, Hypernetwork and Mapper(1–4). In this work, we will focus on Mapper-based TDA.

*Mapper:* Mapper builds a compact representation of the dataset by means of a graph from data. Mapper algorithm consists of a few steps on a point cloud data (PCD) embedded in a metric space (e.g. Euclidean). First, a *filter function is computed.* The function should convey some interesting properties of the data (e.g., the value of a clinical prediction rule). The histogram of the filter function is computed. The points in the PCD are grouped by some similarities (e.g., metric similarities, by biological features) Each group shapes a node of the graph. Given two nodes and the corresponding points, if there are at least two points such that their filter values are in the same bin of the histogram then an edge connecting the nodes is drawn. A more comprehensive review of the technical details of the algorithm is synthesized by the paper by Carlsson et al.(5).

*Topological-Statistical features selection:* the analysis of Mapper graph is completed with statistical analysis for quantifying the differences among topological structures. In details, topological features (i.e., branches and loops) are compared by means of statistical tests (e.g., Kolmogorov-Smirnov, F-test and Chi-squared test). Statistical analysis is used for certifying the statistical relationships among the clusters (nodes in the graph) and to mine the features that differentiate statistically topological substructures. In this work, we used the Chi-squared test to perform a statistical comparison of the topological structures(6).

*Confusion Matrix:* the Confusion Matrix (CM) is a performance measurement for ML classification where output can be represented by two or more classes. It is a table with at least four different combinations of predicted and actual values, as shown in S1 Table. The interpretation is straightforward: we labeled as true positive (TP) the patients correctly predicted as positive by the algorithm, as true negative (TN) the subjects correctly predicted as negative by the algorithm, false positive (FP, or type 1 error) the patients wrongly predicted as positive and false negative (FN, or type 2 error) the subjects wrongly predicted as negative by the algorithm. From the confusion matrix we obtained different accuracy metrics as (i) recall, defined as the ratio between TP/(TP+FN), (ii) precision, defined as the ratio between TP/(TP+FP), (iii) accuracy, defined as (TP+TN)/(total number of predictions), (iv) F-measure, which measures recall and precision at the same time using harmonic mean instead of arithmetic mean to reduce the extreme values.

*Interpretable Machine Learning:* model interpretation can be defined as the ability to understand the decision policies of a ML response function to explain the relationship between input and output variables. Moreover, explanation must be reported in a human readable format. Explanations shall let human to understand the “what, why, and how” of ML algorithmic decisions. Model interpretation is the ability to explain and validate the decisions of a predictive model to enable fairness, accountability, and transparency in the algorithmic decision-making. There are several attempts on extracting interpretation and equipping them with a semantics(7). In this work we focus on two interpretations that are known as “global” and “local” and that are implemented in two Python frameworks, namely Skater and LIME(8,9).

### *Skater*: Skater is a tool that relies on information theory, i.e., entropy, and it is intended to measure the importance of each input feature with regard the prediction outputted by a - machine learning - model. The intuition is that the more a model’s decision criteria depend on a feature, the more we’ll see predictions change as a function of perturbing a feature. Assume one has a dataset with two features respectively f1 and f2 and they are used to train a machine learning model. SKATER assigned score 0.15 to f1 and 0.10 to f2. The user might face two different operational scenarios among the others: 1) f1 is incorrect while f2 is correct, 2) f1 is correct while f2 is incorrect. In the first scenario the machine learning model trained f1 and f2 will have a result less reliable when the same model is used in the scenario number 2.(8).

*LIME:* local interpretable model-agnostic explanations (LIME) is a technique aiming to explain which features are most important in specific areas of the feature space. This can be used to assess -for a specific subject - which features contributed most to its prediction. By collecting statistics from multiple subjects belonging to the same class it is possible to generalize which features contributed most to the prediction of the class(9). In order to show LIME function, we show LIME plots for randomly selected patients: for *TF* we produce three plots, corresponding to class 0, 1 and ICU transfer, respectively. For *stroke/TIA* and *MB* we depict two plots for the two classes. *Therapeutic Failure*: “class 0” represents the group of patients not undergoing TF. The probability for the patient to belong to this class is P(C_0_) = 1. In the selected patient, the features contributing to the success were the absence of aortic valve disease and the absence of AHF (S3 Fig, Panel A). On the contrary, presence of cardiogenic shock and amine use would reduce the probability P(C_0_) to (1–0.18–0.18=0.64). Local interpretation of a patient undergoing in-hospital death (“class 1”) suggests that the variables “no amine use”, “no cardiogenic shock” and “no stroke/TIA” were more relevant (S3 Fig, Panel B), and that their removal would reduce the probabity P(C_1_) to (1–0.1–0.085–0.05=0.49) with a consequence of an immediate misclassification. The LIME graphic in a patient transferred to the ICU (“ICU class”) is quite similar to the one of a patient in “class 1”, with some differences both in the scores and in some values: the removal of “no amine use”, “no cardiogenic shock” and “no stroke/TIA” would cause a reduction of the probability P(C_ICU_) to 0.551 with a high chance of misclassification, underlining the importance of the patient-specific weights for each feature in ML algorithms. *Stroke/TIA:* the analysis of a patient in “class 0” (no stroke/TIA) and of a patient in “class 1” (stroke/TIA) is mainly characterized by “no AHF”, “high TTR”, “no previous stroke/TIA” (S4 Fig, Panels A and B). *Major Bleeding:* the analysis of a patient in “class 0” (no MB) is strongly characterized by the absence of AHF and the absence of acute coronary syndrome (ACS). The patient of “class 1” is characterized “anemia”, “previous gastrointestinal bleeding” and “AHF” (S5 Fig, Panels A and B).

**SUPPLEMENTARY REFERENCES**

1. Rucco M, Sousa-Rodrigues D, Merelli E, Johnson JH, Falsetti L, Nitti C, et al. Neural hypernetwork approach for pulmonary embolism diagnosis. BMC Res Notes [Internet]. 2015 Oct 29 [cited 2015 Oct 29];8(1):617. Available from: http://www.biomedcentral.com/1756-0500/8/617

2. Ray J, Trovati M. A Survey of Topological Data Analysis (TDA) Methods Implemented in Python. In 2018. p. 594–600.

3. Binchi J, Merelli E, Rucco M, Petri G, Vaccarino F. JHoles: A tool for understanding biological complex networks via clique weight rank persistent homology. In: Electronic Notes in Theoretical Computer Science. Elsevier; 2014. p. 5–18.

4. Forsting M. Machine learning will change medicine. Vol. 58, Journal of Nuclear Medicine. Society of Nuclear Medicine Inc.; 2017. p. 357–8.

5. Singh G, Mémoli F, Carlsson G. Topological Methods for the Analysis of High Dimensional Data Sets and 3D Object Recognition. Eurographics Symp Point-Based Graph. 2007;

6. Meyer S, Meyer S. Data analysis for scientists and engineers. 1975 [cited 2020 Jan 18]; Available from: https://fokt.pw/ss.pdf

7. Goebel R, Chander A, Holzinger K, Lecue F, Akata Z, Stumpf S, et al. Explainable AI: The new 42? In: Lecture Notes in Computer Science (including subseries Lecture Notes in Artificial Intelligence and Lecture Notes in Bioinformatics). Springer Verlag; 2018. p. 295–303.

8. Wei P, Lu Z, Song J. Variable importance analysis: A comprehensive review. Vol. 142, Reliability Engineering and System Safety. Elsevier Ltd; 2015. p. 399–432.

9. Ribeiro MT, Singh S, Guestrin C. “Why should i trust you?” Explaining the predictions of any classifier. In: Proceedings of the ACM SIGKDD International Conference on Knowledge Discovery and Data Mining. Association for Computing Machinery; 2016. p. 1135–44.

**S1 Table. Confusion Matrix.** Confusion Matrix for a two-classes problem.

| **Predicted Values** |  | **Actual Values** | |
| --- | --- | --- | --- |
|  |  | **Positive** | **Negative** |
|  | **Positive** | True Positive | False Positive |
|  | **Negative** | False Negative | True Negative |

**S2 Table. Database Structure.** AFICILL Database structure.

| **Variable** | **Format** | **Meaning** |
| --- | --- | --- |
| Admission Date | MM/DD/YYYY | Date of SDU Admission |
| ID | Number | AFICILL ID Number |
| Surname | String | Anonymized in the DB |
| Name | String | Anonymized in the DB |
| Sex | Binary | Sex |
| Age | Number | Age, years |
| Outcome | Categorical | Discharge; ICU Transfer; In-Hospital Death |
| NVAF Type | Categorical | Paroxysmal; Persistent; Permanent |
| Electric Cardioversion | Binary | ECV during the SDU admission |
| Pharmacologic Cardioversion | Binary | PCV during the SDU admission |
| Anticoagulant at Admission | Categorical | Warfarin; LMWH; DOACs; No Anticoagulant |
| Anticoagulant at Discharge | Categorical | Warfarin; LMWH; DOACs; No Anticoagulant |
| Reason for Admission | **Spacer** | |
| Syncope | Binary |  |
| Admitted for Planned ECV | Binary | Planned ECV for NVAF at admission |
| Acute Neurological Syndromes | Binary |  |
| Trauma | Binary |  |
| Thrombosis | Binary |  |
| Haemorrage | Binary |  |
| Acute Coronary Syndrome | Binary |  |
| Acute Heart Failure | Binary |  |
| Cardiogenic Shock | Binary |  |
| Haemorragic Shock | Binary |  |
| Septic Shock | Binary |  |
| Acute Kidney Injury | Binary |  |
| Acute Respiratory Failure | Binary |  |
| Infection | Categorical | Pneumonia; Abdominal Infections; Urinary Tract Infections; Other Infections |
| Comorbidities | **Spacer** | |
| Chronic Heart Failure | Binary |  |
| COPD | Binary |  |
| Peripheral Artery Disease | Binary |  |
| Previous Stroke/TIA | Binary |  |
| Haepatic Pathologies | Binary |  |
| Chronic Kidney Disease | Binary |  |
| Coronary Artery Disease | Binary |  |
| Type 2 Diabetes Mellitus | Binary |  |
| Anaemia | Binary |  |
| Previous Gastrointestinal Bleeding | Binary |  |
| Hypertension | Binary |  |
| Active Cancer | Binary |  |
| Number of Comorbidities | Number | Sum of the chronic comorbidities |
| Labile Time in Therapeutic Range | Binary | TTR according Rosendaal method |
| ASA or Plavix Use | Binary |  |
| Alcohol Abuse | Binary |  |
| Mitral Valve Disease | Binary |  |
| Aortic Valve Disease | Binary |  |
| CHA2DS2-VASC SCORE | **Spacer** | |
| Chronic Heart Failure | Binary |  |
| Hypertension | Binary |  |
| Age | Categorical |  |
| Type 2 Diabetes Mellitus | Binary |  |
| Previous Stroke/TIA | Binary |  |
| Vascular Disease | Binary |  |
| Sex | Binary |  |
| CHADS-VASC | Categorical | CHA2DS2-VASc Score results |
| CHADS2 | Categorical | CHADS2 Score results |
| CHADS-OUTCOME | Binary | In-hospital stroke or TIA |
| HASBLED SCORE | **Spacer** | |
| Hypertension | Binary |  |
| Chronic Kidney Disease | Binary |  |
| Haepatic Pathologies | Binary |  |
| Alcohol Abuse | Binary |  |
| Age | Binary |  |
| Previous Stroke/TIA | Binary |  |
| Previous Major Bleeding | Binary |  |
| Labile Time in Therapeutic Range | Binary | TTR according Rosendaal method |
| ASA or NSAIDS use | Binary |  |
| HASBLED | Categorical | HAS-BLED Score results |
| HAS-OUTCOME | Binary | In hospital occurrence of major bleeding |
| BLOOD EXAMS | **Spacer** | |
| INR | Number |  |
| PLT | Number |  |
| Creatinine | Number |  |
| CKD-EPI | Number | eVFG according to CKD-EPI |
| NOTE | String |  |
| Other Data | String |  |
| SBP | Number | Systolic blood pressure at SDU admission |
| DBP | Number | Diastolic blood pressure at SDU admission |
| ACEi/ARB | Binary |  |
| Diuretics | Binary |  |
| Amine | Binary |  |
| Amiodarone | Binary |  |
| Propafenone/Flecainide | Binary |  |
| Sotalol | Binary |  |
| EF Category | Categorical | Preserved EF; Reduced EF |
| EF | Number | Ejection Fraction |
| APACHE2 | Number | APACHE II Score |

**S3 Table. Selected Features.** Features selected by statistical tests from the topological graph.

| **Therapeutic Failure** | **Stroke/TIA** | **Major Bleeding** |
| --- | --- | --- |
| Sex | Sex | Age |
| Age | Age | Anticoagulant at Admission |
| Anticoagulant at Admission | Electric Cardioversion | Syncope |
| Syncope | Anticoagulant at Admission | Acute Heart Failure |
| Acute Coronary Syndrome | Syncope | Hemorrhagic Shock |
| Cardiogenic Shock | Acute Coronary Syndrome | Acute Respiratory Failure |
| Hemorrhagic Shock | Acute Heart Failure | COPD |
| Septic Shock | CHF | Anemia |
| Acute Respiratory Failure | COPD | Previous Gastrointestinal Bleeding |
| Infection | Previous Stroke/TIA | Labile TTR |
| COPD | Chronic Kidney Disease | ASA or Clopidogrel Use |
| Hypertension | Coronary Artery Disease | Systolic Blood Pressure |
| ASA or Clopidogrel Use | Labile TTR | Diastolic Blood Pressure |
| Mitral Valve Disease | Mitral Valve Disease | ACEi/ARB |
| Systolic Blood Pressure | Aortic Valve Disease | Diuretics use |
| Diastolic Blood Pressure | Systolic Blood Pressure |  |
| ACEi/ARB | Diastolic Blood Pressure |  |
| Amine use | Amine use |  |
| Propafenone/Flecainide use | Amiodarone use |  |
|  |  |  |

**S1 Fig.** **Topological simplification via Mapper algorithm.** Left: 5000 2-dimensional noisy points distributed along two concentric circles; Right: Mapper simplicial complex. Mapper is able to highlight and simplifies the underlined topological shape of the original point cloud data. For the sake of clarity, a circle is topologically equivalent to an ellipse-like shape since they both design a planar hole. Noisy points in the original images cause the flames.


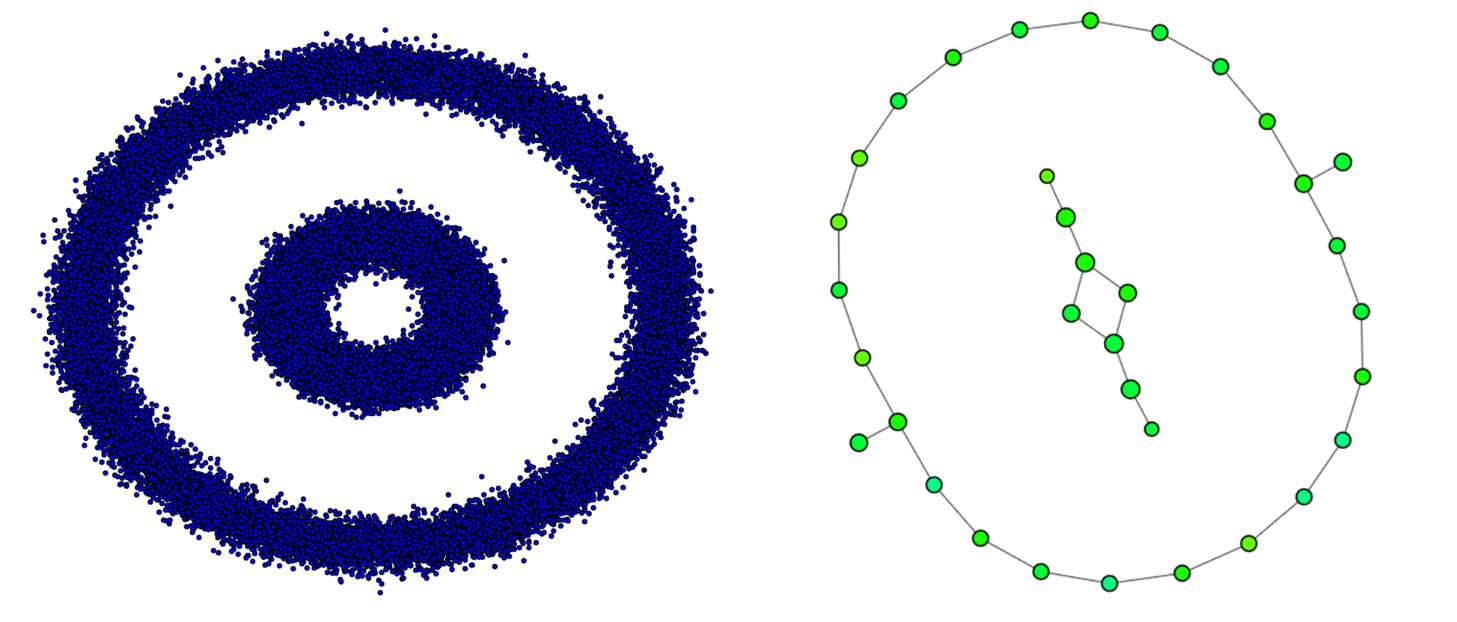


**S2 Fig.** **Classification plots.** Classification plots for ML-derived models for (A) “therapeutic failure”; (B) “stroke/TIA”; (C) “major bleeding”.

| 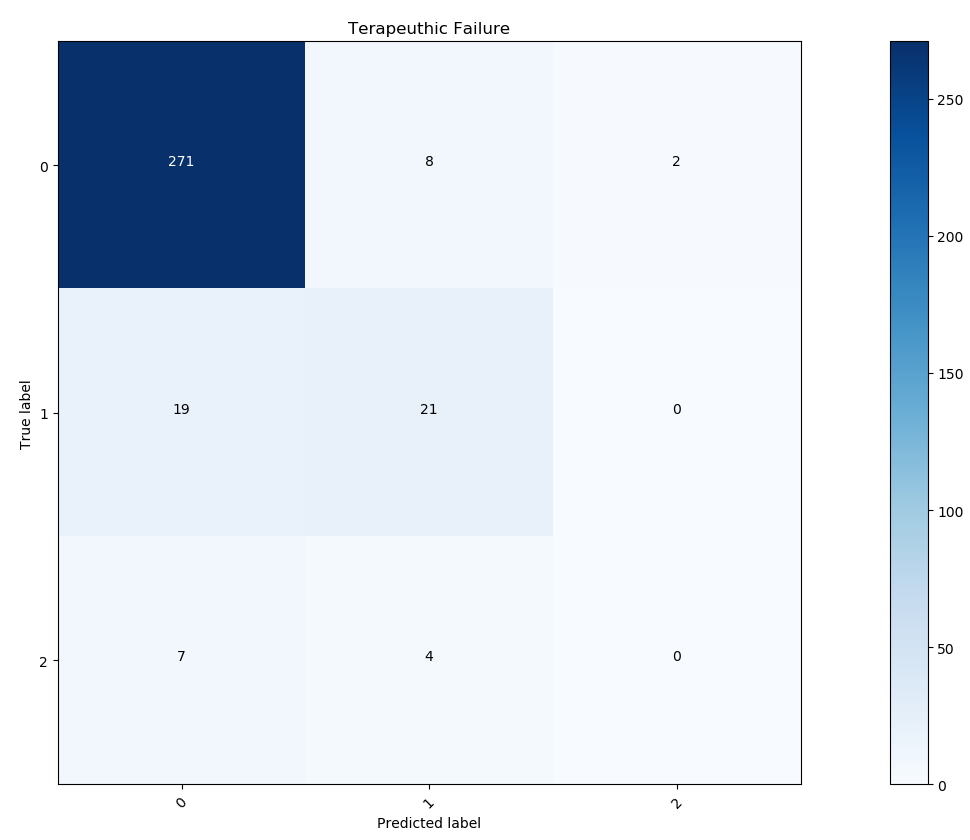A |
| --- |
| 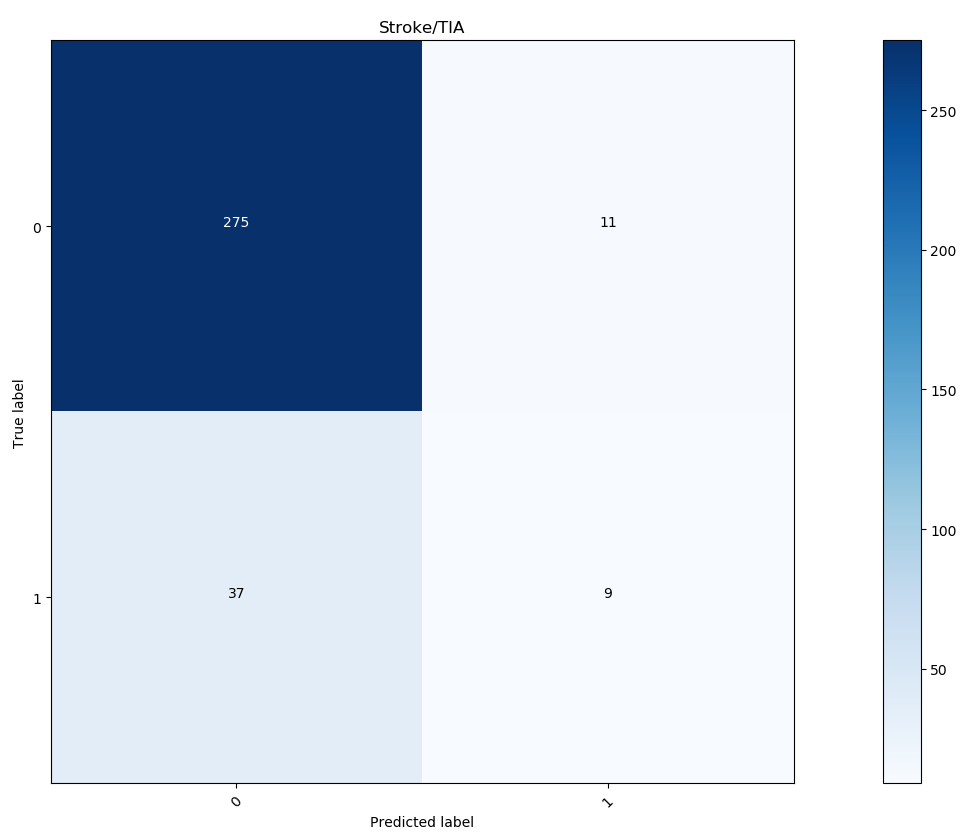B |
| 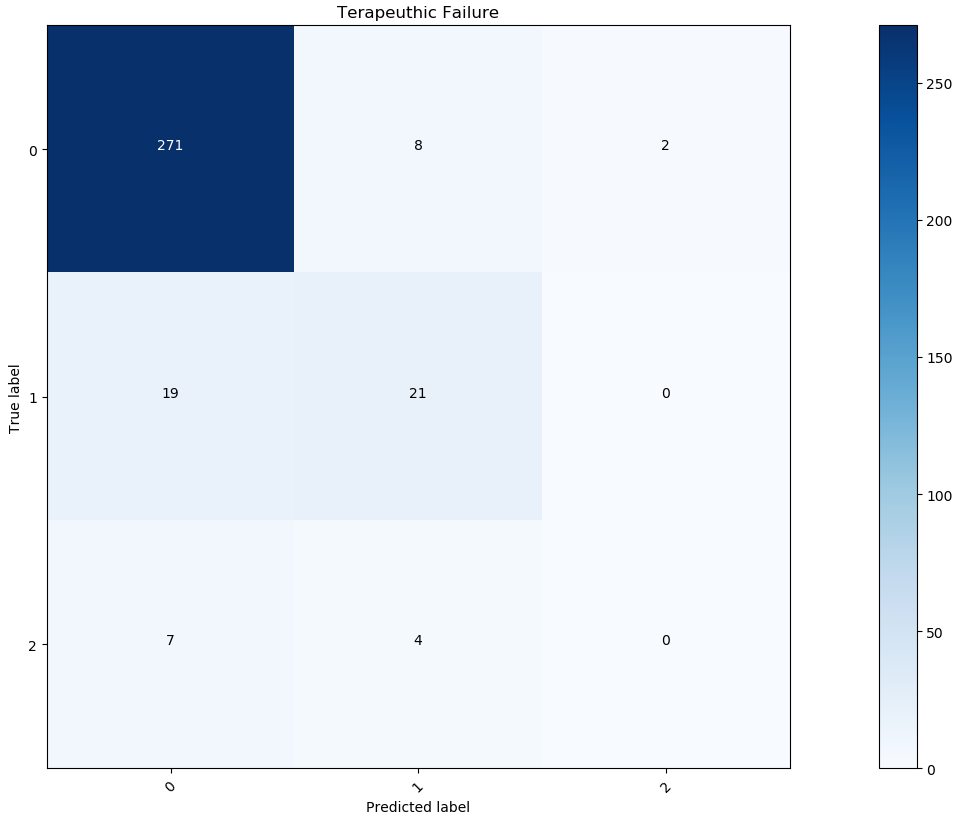C |

**S3 Fig. LIME results.** LIME results for “therapeutic failure” in three patients selected from the cohort.

| A |
| --- |
| B |
| C |

**S4 Fig. LIME results.** LIME results for “stroke/TIA”.

| A |
| --- |
| B |

**S5 Fig. LIME results.** LIME results for major bleeding.

| A |
| --- |
| B |
